# Hysterectomy Sparing Management of Uterine Necrosis following Uterine Artery Embolization for Postpartum Hemorrhage

**DOI:** 10.1155/2023/8276110

**Published:** 2023-07-19

**Authors:** Myriam Chlela, Josette Dawkins, Gregory Lewis

**Affiliations:** ^1^Department of Obstetrics and Gynecology, United Health Services Perinatal Center, 33-57 Harrison Street, Johnson City, NY 13790, USA; ^2^Department of Obstetrics and Gynecology, Baystate Medical Center, 759 Chestnut Street, Springfield, MA 01199, USA; ^3^Department of Obstetrics and Gynecology, University of Florida College of Medicine, 653-1 W. 8th Street, Jacksonville, FL 32209, USA

## Abstract

**Background:**

Postpartum hemorrhage (PPH) is one of the leading causes of maternal morbidity and mortality. Uterine artery embolization (UAE) is an effective procedural intervention for controlling PPH. Uterine necrosis (UN) is a rare complication of UAE and its management usually results in hysterectomy. We highlight a case of UAE complicated by UN managed conservatively without hysterectomy.

**Case:**

This is the case of a 30-year-old patient who had a cesarean section delivery and subsequently developed PPH due to uterine atony. The estimated blood loss (EBL) was 2500 ml; despite the use of uterotonic medications and trial of intrauterine balloon tamponade. She successfully underwent a UAE with no immediate complications. The remainder of her postnatal course was uncomplicated, and she was discharged on postoperative day 4. On postoperative day 28, the patient presented with fever, vaginal discharge, and abdominal pain. An abdomino-pelvic computed tomography scan revealed areas of necrosis within the uterus secondary to recent UAE. After minimal clinical improvement, the patient underwent a dilation and curettage with ultrasound guidance. The patient improved clinically and was discharged home to complete a 14-day course of antibiotics.

**Conclusion:**

UAE is an important minimally invasive approach to the management of PPH. UN following UAE can present a clinical challenge to physicians, with the underlying pathophysiology being use of small embolizing particles during UAE and lack of arterial collaterals to embolized areas. A total of 19 cases of UN post-UAE have been described of which most of these cases were managed with a hysterectomy. In this case, an alternative treatment plan was successfully implemented via dilation and curettage under ultrasound guidance for removal of organized necrotic tissue. This was sufficient to improve the patient's symptoms and clinical outcome and saved the patient from the morbidity and mortality risks associated with a hysterectomy.

## 1. Introduction

Postpartum hemorrhage, defined as a blood loss greater than or equal to 1000 ml after vaginal delivery or cesarean section, is one of the leading causes of maternal morbidity and mortality worldwide [1]. Abnormal uterine tone is estimated to cause between 70% and 80% of postpartum hemorrhage and is often suspected first as the underlying etiology [2]. Procedural modalities to control postpartum hemorrhage often include intrauterine balloons, use of B-Lynch sutures, arterial ligation, and hysterectomy [3]. Uterine artery embolization (UAE) has also evolved as a distinct management option for postpartum hemorrhage and is an excellent uterine sparing alternative when uterotonic agents and intra-cavitary uterine compression balloon devices fail to control the excess bleeding [4]. With the avoidance of surgery and success rates of 88%–100%, UAE has become a necessary and attractive tool in the clinician's armamentarium for managing PPH [5]. UAE does, however, have various associated complications including post-procedure pain, fever, infection, persistent vaginal discharge, non-target embolization, and uterine necrosis. Uterine necrosis represents a very rare adverse outcome of UAE for postpartum hemorrhage; often necessitating a hysterectomy. The clinical features, presentation as well as diagnostic and management guidelines for uterine necrosis post-UAE, are not uniform and have not been well elucidated [6]. We highlight a case of UAE complicated by uterine necrosis and managed without performing a hysterectomy. Herein we report the clinical course of the patient, conduct a brief literature review, discuss etiology, and suggestions for management.

## 2. Case Presentation

**Figure 1:**
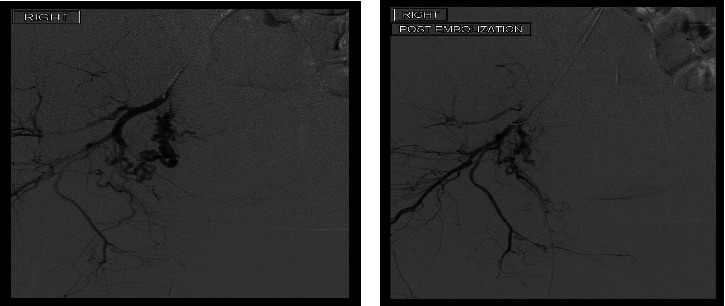
Showing (a) right uterine artery embolization and (b) post-embolization.

**Figure 2:**
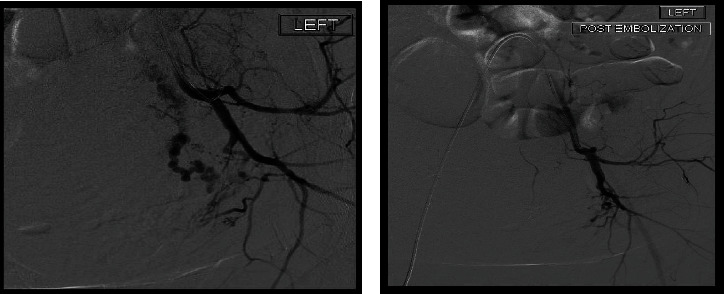
Showing (a) left uterine artery embolization and (b) post-embolization.

**Figure 3:**
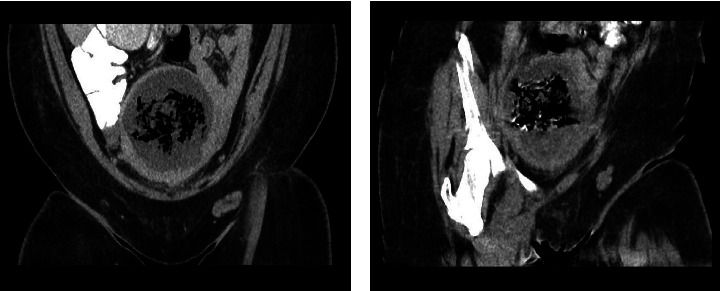
CT scan of the pelvis (a) and (b) showing coronal views of the uterus with areas of necrosis.

**Figure 4:**
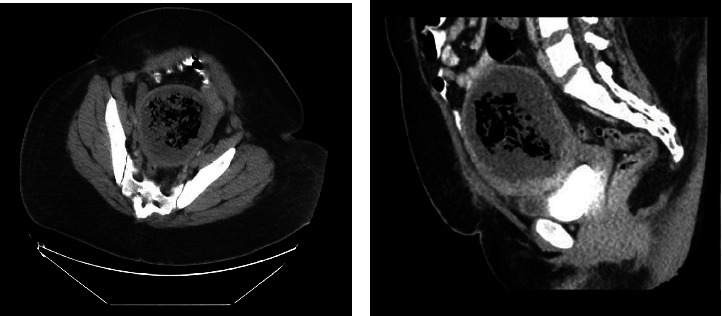
CT scan of the pelvis showing (a) transverse and (b) sagittal views of the uterus with internal foci of gas representing areas of necrosis.

The index case is that of a 30-year-old G4P3013 patient who presented to the labor and delivery unit at 40 weeks and 6 days for induction of labor. Obstetrical history included three previous uncomplicated vaginal deliveries. She had no prior surgical or significant medical history. Her labor induction was started with dinoprostone, which was removed after about 4 hours due to persistent category 2 fetal heart rate tracing. A decision was then made to perform a cesarean section delivery due to the persistent category 2 fetal heart rate tracing, refractory to resuscitative measures. Intraoperative blood loss was estimated at 1000 ml. In the post-anesthesia care unit (PACU), the patient started having increased vaginal bleeding secondary to uterine atony. The bleeding did not respond to uterotonic agents including oxytocin, misoprostol, methylergonovine, and carboprost. Tranexamic acid was also administered; however, this was not effective. We then proceeded with examination under anesthesia and placement of a Bakri balloon was attempted; however, after infiltrating the balloon with normal saline, the device was expelled from the uterus on two occasions. The total estimated blood loss was noted to be 2500 ml, and the patient remained hemodynamically stable. The interventional radiology team was consulted, and the patient underwent bilateral UAE using tris-acryl gelatin microsphere (Embosphere Microspheres^®^) 700–900 microns and Gelfoam slurry (Figures 1 and 2). The patient was discharged on postoperative day 4 after meeting all her postoperative milestones. On postoperative day 28, she presented to the emergency room due to foul smelling vaginal discharge, abdominal pains, fever, and chills. An abdomino-pelvic computed tomography (CT) scan was obtained, which revealed an enlarged uterus with unenhanced areas of the endometrium and myometrium as well as internal foci of gas within the uterine fundus. These findings were suggestive of uterine necrosis, with approximately 50% normal residual myometrial thickness (Figures 3 and 4). She was started on empiric broad spectrum antibiotics. She continued to have persistent malodorous vaginal discharge and remained febrile even after adjusting her antibiotics in consultation with the infectious disease team. The patient declined a hysterectomy as she wanted to preserve her fertility. She was consented for examination under anesthesia, dilation and curettage (D&C) with direct ultrasound guidance 4 days after starting her on antibiotics. The D&C procedure was uncomplicated, and organized necrotic tissue was curetted and sent for pathological and tissue culture evaluation. The pathology results subsequently showed features of acute and chronic endometritis. The tissue cultures grew *Streptococcus anginosus* and *Prevotella* species, and culture directed antimicrobial therapy was commenced. The patient subsequently had improvement in her clinical symptoms—she had no more vaginal discharge and she remained afebrile for more than 48 hours. She was discharged on hospital day 7 to complete a 14-day course of antibiotics. At her follow-up visit, her examination was within normal limits and she denied any abdominal pain, pelvic discomfort, or abnormal vaginal discharge or bleeding and was happy to have retained her uterus. She desired and received an etonogestrel subdermal implant for contraception.

## 3. Discussion

Ischemic complications of UAE, such as necrosis of the uterus or other pelvic structures, are rare. A total of 19 cases of uterine necrosis following UAE have been reported in the literature and do pose a diagnostic and management challenge [7]. The clinical presentation and outcomes of this condition are varied, with 18 out of 19 patients ending up having a hysterectomy. In one case where a hysterectomy was not performed, hyperbaric oxygen was used as the treatment modality [8]. We did not proceed with a laparotomy despite the patient being febrile as she wanted to preserve her uterus, she also remained hemodynamically stable and had no signs of peritonitis. A careful D&C under ultrasound guidance with removal of organized necrotic tissue was sufficient to improve the patient's clinical course and allowed for a safe hospital discharge. This is the first case reported in the English literature where D&C alone under ultrasound guidance was carried out and was successful in decreasing the necrotic burden of the uterus, therefore, improving the patient's clinical status and avoiding a hysterectomy. More radical approaches to management are often adopted for patients where necrosis of other pelvic structures such as the bladder or the adnexa are suspected or in cases where the patient is clinically unstable [6] [9].

Several theories have been proposed explaining the underlying pathology of uterine necrosis post-UAE. First, the size and type of the embolizing particles are important contributing factors. Gelfoam particles less than 500 *μ*m and spherical polyvinyl alcohol particles less than 400 *μ*m can travel far distally and thereby block anastomotic blood flow [5] [9]. The patient in this case had her embolization carried out using tris-acryl gelatin microsphere (Embosphere Microspheres^®^) with size greater than 500 *μ*m. However, the non-absorbable nature of tris-acryl gelatin microsphere as well as polyvinyl alcohol particles makes it more likely for them to reach the distal arterioles and capillaries and thus increasing the likelihood of ischemia [9].

Second, the absence of anastomosis between the uterine artery and the ovarian artery can increase the risk of necrosis, especially in the distended postpartum uterus. Third, the use of high pressure as opposed to free flow embolization has been implicated, as high pressure embolization allows for embolizing agents to reach distal collateral blood vessels and occlude them and thus increases the chance of ischemic necrosis [5] [6]. Other risk factors include advanced age, absence of prophylactic antibiotics, sepsis, and hypovolemic shock [7]. Our patient was of a young age, received prophylactic antibiotics, and remained hemodynamically stable. There have been reports of possible association of uterine necrosis with hypertensive disorders of pregnancy such as pre-eclampsia, especially due to uterine local circulatory failure associated with intravascular volume depletion [10] [11]. The patient in this case did not have this particular risk factor.

The time interval between the embolization procedure and diagnosis of uterine necrosis varies widely and has been reported as early as day 10 to as late as day 53. The time interval seemed to be independent of the embolizing agent used [12] [13]. The patient in this case presented, 28 days after her procedure, with vaginal discharge and fever. No classic presentation of uterine necrosis following UAE has been highlighted in the reported cases. Vaginal bleeding, vaginal discharge, abdominal pains, and fever seem to be the most commonly reported symptoms [5] [7]. Patients who present with features of sepsis are more likely to require exploratory laparotomy and eventual hysterectomy [6]. If early clinical warning signs of uterine necrosis can be ascertained and precautionary measures discussed with patients, this may prompt evaluation and management in a timely fashion and could possibly decrease morbidity.

UAE is an important procedural option for the management of postpartum hemorrhage with less morbidity compared to hysterectomy. Indeed, UAE avoids hysterectomy as a last resort to stop severe bleeding; however, temporary blood vessel occlusion may induce local necrosis and inflammatory reactions, with potential subsequent adverse effects on the endometrium and ovaries, leading to a disturbance of the menstrual cycle and fertility. The patient received a long-acting reversible contraception after discharge; however, discussions regarding future pregnancy and potential outcomes would be prudent as there have been reports of higher rates of preterm birth, fetal growth restriction (FGR), and placenta accreta spectrum (PAS) among women who become pregnant after UAE for PPH [14]. In fact, Gaia et al. reported a pregnancy rate of 62% in patients who desired and attempted conception after UAE [15].

## 4. Conclusion

Generally, hysterectomy is described as a therapeutic strategy for uterine necrosis in the present literature. The patient was successfully spared a hysterectomy and its attendant morbidities in this case, possibly preserving her fertility potential as she requested.

## Data Availability

All information and clinical images related to this case report are included in the article.
